# Metal(loid)s (As, Cd, Cu, and Zn) in three fish species from a dam after a mine-tailing spill: differential bioaccumulation and potential health risk

**DOI:** 10.1007/s10653-023-01509-8

**Published:** 2023-02-28

**Authors:** F. Páez-Osuna, M. E. Bergés-Tiznado, G. Valencia-Castañeda, M. G. Fregoso-López, J. A. León-Cañedo, J. F. Fierro-Sañudo, J. Ramírez-Rochín

**Affiliations:** 1 Instituto de Ciencias del Mar y Limnología, Unidad Académica Mazatlán, Universidad Nacional Autónoma de México, Joel Montes Camarena s/n, Playa Sur, P.O. Box 811, 82040 Mazatlán, Sinaloa Mexico; 2 Miembro de El Colegio de Sinaloa, Antonio Rosales 435 Poniente, Culiacán, Sinaloa Mexico; 3 Unidad Académica de Ingeniería en Tecnología Ambiental, Universidad Politécnica de Sinaloa, Carretera Municipal Libre Mazatlán-Higueras Km. 3, C.P. 82199 Mazatlán, Sinaloa Mexico; 4 Instituto de Ciencias del Mar y Limnología, Universidad Nacional Autónoma de México, Av. Ciudad Universitaria 3000, Coyoacán, 04510 Ciudad de Mexico, Mexico; 5 Facultad de Ciencias del Mar, Universidad Autónoma de Sinaloa, Paseo Claussen s/n Col. Centro, 82000 Mazatlán, Sinaloa Mexico; 6 Universidades para el Bienestar Benito Juárez García-Sede Etchojoa, localidad Buaysiacobe, Etchojoa, Sonora Mexico

**Keywords:** *Cyprinus carpio*, Gulf of California, Heavy metals, *Micropterus salmoides*, *Oreochromis aureus*

## Abstract

The failure of a tailings dam occurred in January 2013 at the Santa María de Otáez mining region (Mexico) released a spill (~ 300,000 m^3^) on Los Remedios River, which was transported through the San Lorenzo River, and finally to El Comedero (EC) dam. The concentrations of metal(loid)s in the muscle, liver, gills, and guts of three fish species (*Cyprinus carpio*, *Oreochromis aureus*, *Micropterus salmoides*) collected from EC dam were examined twenty months later to assess the performance of the cleaning operations. The bioaccumulation patterns of the metal(loid)s in the tissues were different in the three fish species. Tilapia had the highest Cd (11.23 ± 8.53 µg g^−1^) and Cu (871 ± 1261 µg g^−1^) concentrations in the liver, as well as As concentration (83.6 ± 61.7 µg g^−1^) in the gut, while the highest Zn concentration (745 ± 356 µg g^−1^) was measured in the gills of the carp. Such variability can be caused by the variant bioavailability of the metal(loid)s and by the feeding habits of each species; and also by the fact that some metals are essential and better regulated by organisms than other non-essential. Compared to a study of tilapia carried out 90 days after mine spill, a decrease was evident in the liver for As, Cd, Cu, and Zn by 129, 5, 10, and 1.7 times, respectively. This revealed that cleaning operations were more efficient for As. The target hazard quotient and the hazard index were < 1, which indicates there will be no risk of consuming muscle in moderated rations of the three fish species.

**Supplementary Information:**

The online version contains supplementary material available at 10.1007/s10653-023-01509-8.

## Introduction

Past and present mining activities are among the most common sources of highly toxic chemical substances in aquatic and terrestrial ecosystems. The discharge of large quantities of materials occurs either directly from milling plants, or indirectly through accidental impoundment failures. Tailings are among the main wastes that are often stored in impoundments behind dams, which can fail and have subsequent environmental, economic, and human health impacts (Kossoff et al., 2014). The mining industry is expected to grow in the coming decades, following the trends in metal demand, which has increased due to a growing world population and high per capita requirements (Elshkaki et al., 2016). These trends can cause a serious problem concerning the disposal and management of an increasing volume of waste generated by the mining industry. Besides, poorly constructed and/or heavily charged dams eventually lead to the rupture of reservoirs, resulting in frequent (two or five per year in the world) accidents that spread pollutants into vast areas (Kossoff et al., 2014). The chemical composition of tailings from mining activities depends on the mineralogy of the ore body, nature of the processing fluids, efficiency of the extraction process, and the degree of weathering during storage in the dam impoundment. Metal(loid)s are present in tailings since no extraction reaches complete efficiency; As, Cd, Cu, and Zn, among other elements, generally exhibit high concentrations (Kossoff et al., 2014).

In Mexico, mining is a traditional economic activity mainly dedicated to the production of Cu, Zn, Pb, Ag, Fe, and Au. On the continental margin of the Gulf of California, numerous sites of mining interest were or are being exploited (Páez-Osuna et al., 2017). Therefore, the frequency of spills associated with mine tailings and dam failures in the Gulf of California eco-region is relatively high. Between 2013 and 2021, at least nine accidents of different magnitudes (10,800–300,000 m^3^) have occurred (Páez-Osuna et al., 2022). Most of them originated on the continental margin of the Gulf. Except for the accidents of Cananea (Sonora) and Santa María Otáez (Durango), no other studies were carried out to assess the environmental impact and eventually implement any remediation for the remaining spills.

**Fig. 1:**
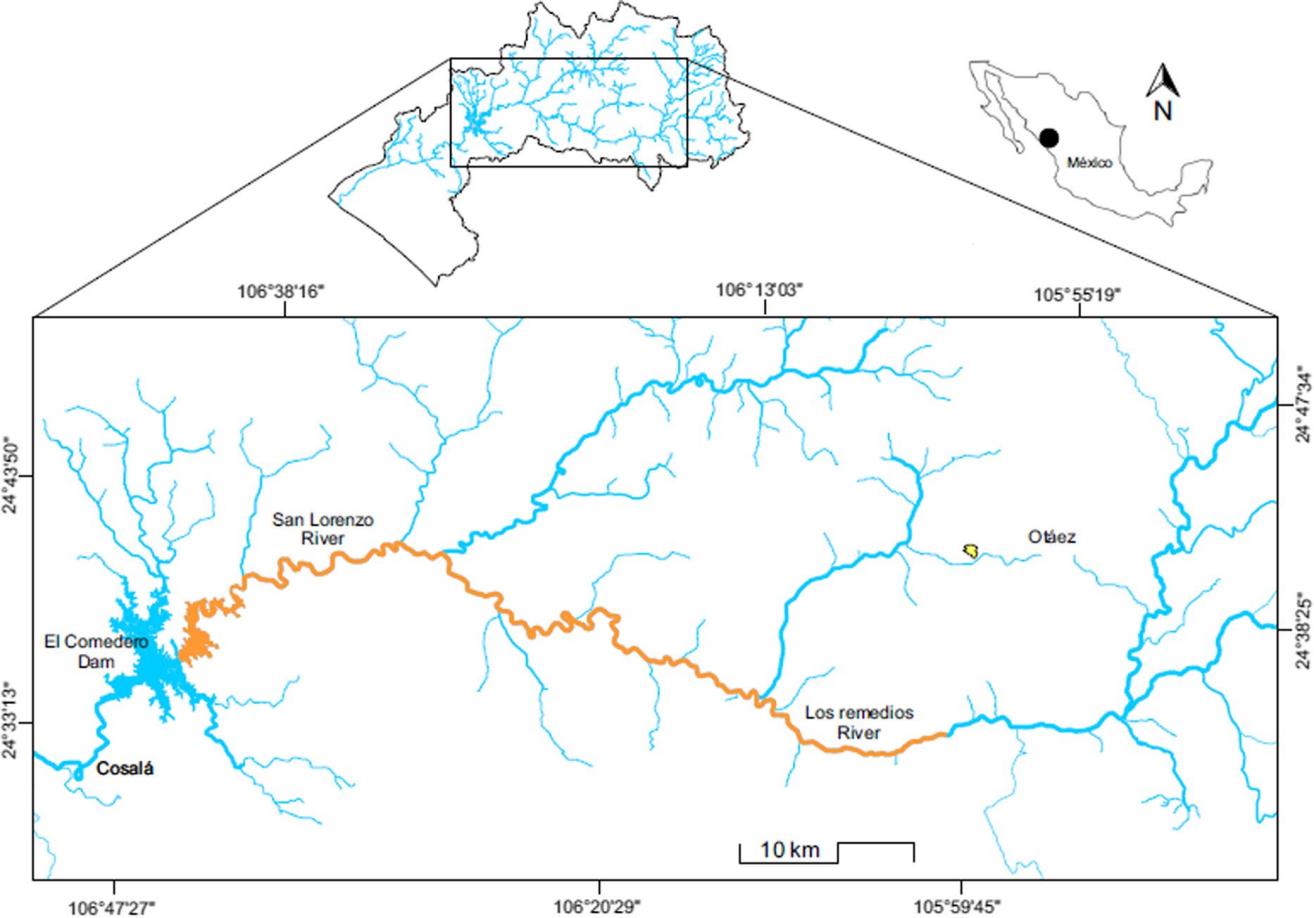
Illustration of the spill-affected zone along the Los Remedios River-Upper San Lorenzo River-El Comedero dam (orange color); right extreme corresponds to the discharge site where the mine tailings and dam failure occurred

In January 2013, a mine spill (~ 300,000 m^3^) affected Los Remedios (LR) River (a tributary of San Lorenzo River), upper San Lorenzo River, and El Comedero (EC) dam (Fig. 1). This had a major impact on the waters and suspended sediments (Páez-Osuna et al., 2015), and it also caused massive fish mortality (Páez-Osuna et al., 2022). In September 2014, twenty months after the mine spill (~ 17 months after the massive fish mortality), the situation changed in the spill-affected section (Fig. 1) of the San Lorenzo River and EC dam. An emergency soil clean-up procedure was implemented after the accident during a short term (1–2 weeks), which consisted simply in remove mechanically the sludge covering the site of discharge of LR River. Even with those clean-up operations, the affected zone could exhibit high/residual concentrations of metal(loid)s. Thus, twenty months after the mine spill, we collected a set of samples from the EC dam of three fish species to assess the bioaccumulation of As, Cd, Cu, and Zn in the muscle, gill, liver, and gut of the common carp *C. carpio*, the blue tilapia *O. aureus,* and the largemouth bass *M. salmoides*. We tested the hypothesis that three fish populations with different feeding habit exhibit variable accumulation of metal(loid)s in an ecosystem previously affected by a mine-tailing spill. A second hypothesis is that twenty months after mine spill (17 months after massive mortality) a reduction of the metal(loid) concentrations in fish should be reached; and, finally, this reduction must be sufficient so that the muscle does not represent a risk to the health of consumers. Therefore, this study has the following aims: (1) to determine the concentration of metal(loid)s in the three fish species to evaluate the differences among fishes and tissues; (2) to assess the performance of cleaning operations and pollution status through the blue tilapia *O. aureus* growing in the spill-affected dam twenty months after the accident, and to compare these results with those obtained from the blue tilapia during the massive mortality event that occurred 3 months after the mine spill (Páez-Osuna et al., 2022); and (3) finally, the potential health risk associated with human consumption of muscle of these three fishes was estimated.

## Materials and methods

### Study area

El Comedero dam, located (24° 30′ N; 106° 45′ W) in the southeastern Gulf of California (Mexico), has a surface of ~ 9200 ha and a volume from 400 to 1900 Mm^3^ (Fig. 1). The dam can reach a depth of 70 m and surficial water temperature ranges from 21.9 °C in January to 31.2 °C in June. It receives waters from the upper San Lorenzo River, which is formed in the Sierra Madre Occidental. The common carp *C. carpio*, the largemouth bass *M. salmoides*, and the blue tilapia *O. aureus* were introduced in EC dam for economic, alimentary, and tourism purposes (Beltrán Álvarez et al., 2015), tilapia being the main inhabitant of this dam.

### Sampling and chemical analysis

A total of 45 fishes were collected in EC dam (Fig. 1): *C. carpio* (*n* = 7), *M. salmoides* (*n* = 22), and *O. aureus* (*n* = 16). Each specimen was measured, weighed, and dissected to separate the liver, gills, guts, and a portion of muscle (Table 1). Due to the limited availability of carp, only seven specimens were worked. Finally, the separated fish tissues were preserved in a freezer (−20 °C) for subsequent laboratory analysis. The guts with their content were examined to evaluate the metal(loid)s levels in the diet of the fish. The separated tissues of the three fish species were lyophilized (72 h, − 52 °C and 75 × 10^−3^ mbar), pulverized, and homogenized in a semiautomatic agate mortar. Acid digestion (5 mL of concentrated (~ 70%) nitric acid, Instra-analyzed J.T. Baker) of duplicate aliquots (0.250 g) was carried out using Teflon vials (Savillex) at 125 ºC for 3 h (Bergés-Tiznado et al., 2015). Only livers were digested using 2 mL of H_2_O_2_ (30%) and 3 mL of concentrated HNO_3_. Analyses of tissue samples were made by AAS. Arsenic was analyzed by AAS with a Zeeman correction background effect coupled to a graphite furnace oven (AAnalyst 800, Perkin-Elmer). A matrix modifier, a solution of Pd(HNO_3_)_2_ and Mg(NO_3_)_2_, was used in each sample atomization for this metalloid. To assess the accuracy of the employed procedure, a reference material for fishes DOLT-4 (dogfish liver) NRC-CNRC (2008) was analyzed. Concentrations of the analyzed elements were within the certified values (recoveries 92.2–105.9%, Table 1S). Precision fluctuated from 0.7% for Cu to 5.2% for Cd. To assess contamination, one blank for every 10 samples was analyzed using this procedure.

**Table 1:** Morphometric variables of species caught in El Comedero dam

Species	Diet	Total length (cm)			Weight (g)		
		Min	Max	Mean ± SD	Min	Max	Mean ± SD
*C. carpio* (*n* = 7)	O1	36.5	47.0	40.3 ± 4.1^c^	625	1725	1002 ± 400^c^
*O. aureus* (*n* = 22)	O2	21.0	34.0	26.3 ± 2.7^a^	165	670	315 ± 102^a^
*M. salmoides* (*n* = 16)	P	24.0	38.0	31.9 ± 4.1^b^	170	740	490 ± 181^b^

*O1* Omnivorous but feed primarily on plants, *O2* Omnivorous (benthic) but also ingest detritus, *P* Piscivore (benthopelagic), *SD* Standard deviation, Different superscript letters indicate significantly different (*p* < 0.05) mean values between the variables of each species

### Risk assessment

The non-cancer risk assessment was calculated as the individual target hazard quotient (THQ) and the sum of THQs as the hazard index (HI) by comparing and estimate of exposure to a reference dose (RfD) for oral exposures (EPA, 2005): THQ = [EF × ED × FIR × C/RfD × BW × AT] × 10^–3^ and HI = ΣTHQ, where EF is an exposure frequency of 365 days year^–1^, ED is a 70-year exposure period, C is the mean concentration of the element (mg kg^−1^), BW is the population body weight of 75, 65 and 20 kg for adult men, female and children (3–5 years old), respectively, AT is the average exposure of 25,500 days and FIR means the food ingestion rate under two different scenarios of consumption. One according the specific fish species and the second under an intake ration of 200 g week^–1^ (28.6 g day^–1^) equal to the total fish consumption rate per capita of Mexico in 2020 (SEMARNAT, 2021). The FIR considered for each species was for blue tilapia 15 g week^–1^ (2.2 g day^–1^), common carp 3 g week^–1^ (0.4 g day^–1^) and the largemouth bass 6.3 g week^–1^ (0.9 g day^–1^). There will be risk if THQ or HI > 1, the RfD data for As, Cd, and Zn were taken from the IRIS Assessment Base (EPA, 2022). It is important to indicate that the As average level was considered as inorganic As (Asi) to be conservative about risks; also, Cu has not been evaluated. Finally, a safe intake was calculated according to the Provisional Tolerable Intake (PTI) per body weight (BW) set by the Joint FAO/WHO Expert Committee on Food Additives (JECFA). The data for each element were (WHO, 2022): Cd 25 μg kg^−1^ BWmonth^−1^; Cu 0.5 mg kg^−1^ BW day^−1^; and Zn 0.3 mg kg^−1^ BW day^−1^. The PTI for As was withdrawn given the last data was considered no longer protective, with a best estimation exposure of 0.1–3 μg kg^−1^ BW/day for Asi. Thus, the lower limit range was used to evaluate the risk (0.1 μg kg^−1^ BW day^−1^).

### Statistical analysis

The databases were done in Excel and the variables were tested using STATISTICA (version 7, StatSoft Inc.). The data were normally distributed and homoscedastic. The results were statistically compared between tissues, species, elements and molar ratios by a one-way ANOVA and Tukey post-hoc tests. The associations or correlations established among the variables were assessed by a Product-Moment Correlations test yielding an r statistic.

## Results and discussion

On January 21, 2013, the dam of El Herrero processing plant, in the Santa Maria de Otáez mining region, NW Mexico, suffered a rupture and released ~ 300,000 m^3^ of mining tailing into RR (Fig. 1) (Páez-Osuna et al., 2015). The tailing spill occurred ~ 150 km away from EC dam. Considering the current, sinuosity, and topography of LR (a tributary of the upper San Lorenzo River that flows into EC dam), the material spilled was probably transported in a period of ~ 35 days. Afterward, the transported material was accumulated at the entrance of EC dam, slowly increasing the volumes of mine tailings in such a way that fishes were exposed to various metal(loid)s causing massive (~ 3000 kg) fish mortality ~ 90 days after the mine spill occurred; The mortality of fish could have occasioned from the combined effect of the metal(loid)s, as well as other residues present in mine tailings (Páez-Osuna et al., 2022). This study was executed twenty months after the mine spill (~ 17 months after the massive fish mortality). During the subsequent 3–5 weeks of the spill, the situation changed on the spill-affected section of RR; a clean-up procedure was implemented and the sludge was removed from most of the affected land. However, the affected zone could exhibit some pollution by metal(loid)s even with the clean-up operations.

### Metal(loid)s in fish tissues

The specimens of the three fish species examined exhibited variable sizes (Table 1); however, they correspond to pre-adults and adults. It is known that fish morphometrics and sex can influence metal(loid)s tissue concentrations (Bergés-Tiznado et al., 2015, 2021; Páez-Osuna et al., 2022; Phillips, 1980). Moreover, few samples were available for the common carp (*n* = 7); then, it is assumed that results and comparisons are interpreted with caution because of that variability. However, here is made a robust description of the variability of metal(loid)s concentrations considering particularly the tissues of the three fish species considering their different feeding habits.

**Fig. 2:**
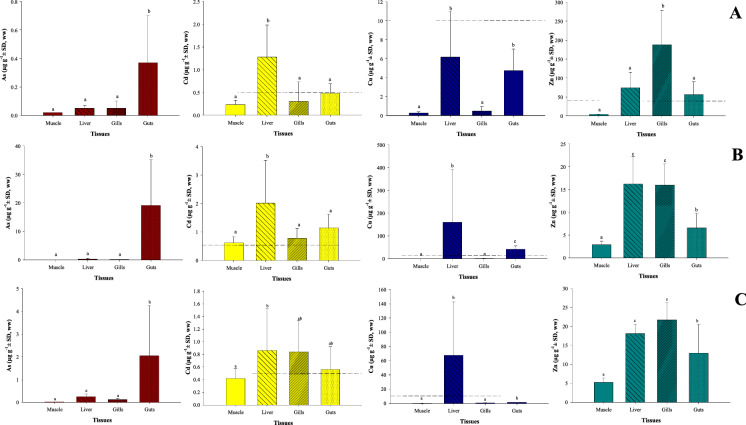
Concentrations of As, Cd, Cu, and Zn (µg g^−1^ ± SD, ww) in tissues of fish species: **A** common carp; **B** blue tilapia; and **C** largemouth bass. SD = standard deviation; different superscript letters indicate significantly different (*p* < 0.05) mean concentrations between tissues of each species

In general, the sequence of the element concentrations was different among tissues and fish species (Fig. 2). The sequence was equal only in the liver of the tilapia and the largemouth bass (Cu > Zn > Cd > As), as well as in the muscle of the carp and bass (Zn > Cu > Cd > As). In the remainder tissues of the three fishes, the sequence was similar, with the highest concentrations of Zn and/or Cu and the lowest of Cd or As (Fig. 2). Metal(loid) concentrations exhibited great variability in the tissues, particularly in the liver and guts of the three fish species, being most remarkable in the guts of the common carp and blue tilapia. Such variability could be explained by the different habitats and different feeding habits of each species, as well as by the variable metal(loid) exposure associated with each habitat occupied by each fish species; the common carp is predominantly omnivorous but feeds primarily on plants; the blue tilapia is omnivorous (benthic) but also ingests detritus; and the largemouth bass is a benthopelagic piscivore.

Common carp: The As mean concentration in the guts was significantly higher (*F* = 6.9, *p* < 0.05) than in the rest of the analyzed tissues, ranging from 0.08 to 0.99 µg g^−1^ wet weight (ww). Conversely, Cd concentration in the liver was higher (*F* = 8.8, *p* < 0.05) than in the guts, muscle, and gills (Fig. 2). The pattern followed by Cu was similar to Cd, with higher averages in the liver. However, it exhibited similar mean Cu levels in the guts and the lowest in the muscle. Zinc concentration was higher in the gills (*F* = 15.2, *p* < 0.05) compared to the rest of the studied tissues, followed by the liver, guts, and muscle (Fig. 2). The morphometric variables of total length (TL) and weight in the common carp were not significantly correlated (*p* > 0.05) to the measured elements in the studied tissues, except for Cu in the liver, which showed a tendency to decrease as the organism increased its TL and weight (Fig. 1SM).

Blue tilapia: The levels of As, Cd, and Cu in the blue tilapia followed the same tendency as the common carp, with significantly higher levels of As in the guts (*F* = 30.2, *p* < 0.05) in comparison with the rest of the tissues, and the highest Cd levels in the liver (*F* = 12.6, *p* < 0.05). The Cu found in the liver of the blue tilapia was higher (*F* = 11.9, *p* < 0.05) than in the guts, gills, and muscle. The content of Zn found in the liver of the tilapia was higher (*F* = 57.0, *p* < 0.05), but statistically there is no difference as the mean concentration found in the gills, followed by the guts; the lowest Zn concentration was found in the muscle (Fig. 2). None of the biometric data of the blue tilapia was significantly correlated (*p* > 0.05) with the measured elements in the studied tissues.

Largemouth bass: The highest As mean concentration (*F* = 12.2, *p* < 0.05) was found in the guts with a range between 0.19 to 9.08 µg g^−1^ (ww), followed by the liver, gills, and muscle (Fig. 2). Significant differences were found between Cd concentration averages in the studied tissues (*F* = 3.6, *p* < 0.05); the highest was in the liver, but they were similar in the gills, and guts, while the lowest Cd concentration was found in the muscle. Moreover, the highest Cu levels were found in the liver, followed by guts, gills, and the lowest in muscle. The means of Zn concentration in the gills were significantly higher (*F* = 37.7, *p* < 0.05) than in the guts and muscle, but the mean Zn concentration in the gills was comparable (*p* > 0.05) with the liver. The size (TL) of this fish was significantly correlated to As and Cd concentrations in the muscle and gills, as well as Zn concentration in the guts. Furthermore, the same significant correlations (*p* < 0.05) were found between the weight and As concentration in muscle and gills, as well as Cd concentration in muscle and gills; weight was also negatively correlated to Zn (*p* < 0.05) concentration in the gills and guts (*p* < 0.05) (Table 2SM).

The accumulation pattern in the tissues was different in the fish species and by metal(loid)s. In the three fish species, Cd and Cu were consistently higher in the liver than in other tissues, while Zn concentration showed a different pattern; in the tilapia, the liver had the highest level, while the carp and largemouth bass exhibited higher concentrations in the gills. Arsenic showed a different pattern that was consistent in the three fish species; the lowest concentration was observed in the muscle and the highest in the guts (Fig. 2). In general, this last pattern corresponds with some previous studies in freshwater (Yap et al., 2015) and marine (Páez-Osuna et al., 2017; Ruelas-Inzunza et al., 2011, 2020) fish species related to various types of organ exposures to the contaminated aquatic environment in the dam as well as the organ specificity regarding the uptake, storage, regulation, and excretion abilities (Bergés-Tiznado et al., 2015) of each species. The high accumulation of Cd, Cu, and partially Zn in the liver is directly associated with metabolism and respiration (Páez-Osuna et al., 2017), given that diet and water are the main routes of capture and assimilation. The liver`s ability to accumulate these elements is a result of the activity of the metallothioneins, which interact with these elements reducing their toxicity (Yap et al., 2015). The metallothioneins induction in fish is well known to be high directly in tissues involved in metal(loid) uptake, storage, and excretion, such as the liver and kidney (Páez-Osuna et al., 2017; Viarengo et al., 2007). Other factors related to the high accumulation of metal(loid)s and metallothioneins in fish are the exposure time and the metal(loid) concentration.

The liver of the three fish species is highly active organ in the uptake, storage, and detoxification of metals, particularly of Cd and Cu; therefore, this organ has been considered a potential biomonitor of metal pollution since liver concentrations are proportional to those present in the environment (Yap et al., 2015). Considering the metal(loid)s levels in the liver of the blue tilapia examined during the massive mortality event occurred three months after the mine spill (scenario 1: Cd 52.0 ± 24.6, Cu 8758 ± 3692, Zn 220 ± 50 and As 200 ± 75 µg g^−1^ dw) (Páez-Osuna et al., 2022) versus the ones found in this study (20 months after the mine spill) (scenario 2: Fig. 2), the reduction in the concentrations is evident for the four metal(loid)s: As 129.0 ± 62.5, Cd 4.6 ± 2.2, Cu 10.1 ± 4.2, and Zn 1.7 ± 0.4 times. This indicates that the performance of the cleaning operations and the natural depuration was more efficient for As and less for Cu, Cd, and Zn. The baseline levels in the blue tilapia are not available in the study area; however, there are experimental studies indicating that liver in control organisms accumulates 0.19 µg Cd g^−1^ (Allen, 1995). Therefore, the concentrations found (Cd 11.2 ± 8.5 µg g^−1^) twenty months after the mine-tailing spill are yet higher (~ 59 times). During 2004–2005, Frías-Espericueta et al. (2010) examined the concentrations of Cd, Cu, and Zn of blue tilapia in El Salto dam, a close (~ 30 km) reservoir which is affected by surface runoff from the nearby agricultural areas; the concentrations found in the liver were 0.71 ± 0.31 for Cd, 147 ± 67 for Cu, and 38.9 ± 12.1 µg g^−1^ (dw) for Zn. These concentrations are lower ~ 16, ~ 6, and ~ 2 times, respectively, than those found in our study for EC dam twenty months after the mine spill.

The guts and their content reflect the recent uptake of the diet consumed by fishes during the last hours before specimens are collected. In the present case, the concentrations of As, Cd, and Cu were more elevated in the blue tilapia than in the other fishes, except for Zn. This can be explained by the different feeding habits of the three species; the tilapia is an omnivore and eventually captures its food (detritus) from the sediments, the carp is omnivorous but feeds primarily on plants, and the largemouth bass is a piscivore. Once ingested, metal(loid) uptake occurs in the intestines through membranes via transporter proteins or/and ionic channels (Le Croizier et al., 2018). Thus, dietary accumulation first takes place in the digestive tract. After reaching the liver, metal(loid)s are released into the general blood circulation and finally reaches secondary accumulation organs, such as the muscle. Conversely, metal(loid)s in fishes are depurated mainly through urine in the kidney and bile excretion from the liver to the intestine, before final elimination through feces (Le Croizier et al., 2018).

Although the existence of size and dependent metal accumulation by aquatic biota is well known from early studies (Phillips, 1980, and references therein), the explanation of this behavior for each element is difficult to describe. The size effect may be a function of any one or several age-dependent parameters. It may depend on differences between the surface/volume ratio, as well as the metabolic and feeding rates of larger (older) and smaller (younger) individuals (Páez-Osuna et al., 1995). The most frequent is that this behavior has been associated with the feeding habit differences between older and younger individuals (Páez-Osuna et al., 1995). Nevertheless, an evident tendency for accumulation of Cu was observed in the livers of carps that belonged to smaller organisms (Fig. 1SM). Similarly, the accumulation of Zn in guts and As in muscle of the largemouth bass was evidenced. In contrast, a higher accumulation of As in gills, Cd in muscle, Cd in gills, and Cd in the guts was found in the largemouth bass (Fig. 2).

### Comparison with other regions

Common carp: a comparison of the concentrations of the metal(loid)s in the tissues of the common carp found in this study with those reported from other areas was carried out (Table 2). The highest levels of the four elements were generally found in livers, while the lowest in muscle, which is a pattern observed for a wide spectrum of fish species (Páez-Osuna et al., 2017). It is noticeable that As in muscle (0.09 ± 0.04 µg g^−1^ dw) from this study exhibited similar concentrations to those reported in most of the regions with activities associated with agriculture, industry, mining, and urbanism (Table 2). Cadmium in muscle (1.07 ± 0.27 µg g^−1^ dw) and liver (4.45 ± 3.52 µg g^−1^ dw) from this study showed the highest levels compared to those reported in several regions of the world (Table 2). Copper and Zn in both tissues of this study showed intermediate concentrations compared to those reported for different types of polluted areas (Table 2). From these results, it is evident that the carp in EC dam is exposed to higher levels of Cd, while the rest of the elements evidenced comparable levels.

**Table 2:** Ranges and mean concentration (µg g^−1^ dw) of arsenic, cadmium, copper, and zinc in the common carp from around the world

Tissue	As	Cd	Cu	Zn	Type of pollution	Region	References
Muscle	–	0.72 ± 0.01	–		Exposure: 30 days 100 µg L^−1^ CdCl_2_	Experimental	Rajeshkumar et al. (2017)
Liver	–	0.93 ± 0.04	–	–			
Muscle	–	0.9 ± 0.1	15.9 ± 7.8	46.9 ± 5.9	Agriculture and aquaculture	Keban Dam lake, Turkey	Danabas et al. (2020)
Liver	–	3.1 ± 0.5	38.2 ± 7.3	301.8 ± 11.4			
Muscle	–	0.02 ± 0.04	7.92 ± 7.99	120.9 ± 106.2	Agriculture and farmed fish	Alagol wetland, Iran	Zafarzadeh et al. (2018)
Liver	–	1.57 ± 0.2	72.6 ± 3.42	519 ± 44	Gradient of pollution (urban)	Flanders river system, Belgium	Delahaut et al. (2019)
Muscle	0.70–2.90	0.28–0.82	0.63–2.01	104–305	Agriculture, industrial and urbanism	Mangla lake, Pakistan	Saleem et al. (2021)
Muscle	–	–	0.05–0.23	0.19–0.97	Industrial	River Swat, Pakistan	Alam et al. (2002)
Liver	–	–	3.2–11.4	26.9–80.3			
Muscle	–	2.27	1.6	24.3	Agriculture	Karacaoren dam, Turkey	Kalyoncu et al. (2012)
Muscle	0.08	0.28	4.35	11.5	Electronics, mining industry	Nansi lake, China	Zhu et al. (2015)
Muscle	0.166	0.011	2.46	271	Urbanism	Chaohu lake, China	Fang et al. (2017)
Muscle	0.095	0.009	0.249	5.43	Agriculture, industrial, urban	Lake Kasumigaura, Japan	Alam et al. (2002)
Muscle	0.044–0.193	< 0.004	0.36–2.39	8.8–16.0	Agriculture and suburban area	Chapala lake, Mexico	Alvarado et al. (2021)
Liver	0.038–0.126	0.013–0.472	6.4–47.8	38.4–265.0			
Muscle	0.09 ± 0.04	1.07 ± 0.27	1.26 ± 0.51	19.2 ± 4.2	Mining tailing spill (after of 20 months)	El Comedero dam, NW Mexico	This study
Liver	0.15 ± 0.07	4.45 ± 3.52	18.6 ± 12.3	264 ± 228			

– Not analyzed, moisture levels considered to change from wet weight to dry weight, muscle 83.2%, liver 80.5%, and guts 74.3% in viscera

Blue tilapia: In general, the four metal(loid)s showed that the highest levels were found in livers and the lowest in the muscle, a pattern observed for a wider variety of species of tilapias. Arsenic in muscle (0.09 ± 0.07 µg g^−1^ dw) and liver (1.55 ± 1.20 µg g^−1^ dw) from this study showed comparable or low concentrations compared to the compiled studies (Table 3), in which levels have been associated with groundwater and several activities such as textile, agriculture, and urban sewage. It is noticeable that Cd concentrations in muscle (3.10 ± 0.94 µg g^−1^ dw) and liver (11.2 ± 8.5 µg g^−1^ dw) of *O. aureus* from this study exhibited higher concentrations than those reported for several tilapia species from polluted regions (Table 4). In our case, Cu (1.48 ± 0.49 µg g^−1^ dw) and Zn (14.6 ± 4.1 µg g^−1^ dw) in the muscle showed low or intermediate concentrations compared to those registered in several tilapia species associated with polluted areas (Table 4). Similarly, Cu (871 ± 1261 µg g^−1^ dw) and Zn (89.6 ± 33.8 µg g^−1^ dw) concentrations in the liver from EC dam were high or comparable to Cu levels in those observed in polluted regions (Table 4). Clearly, these tendencies showed that the blue tilapia in EC dam is exposed to higher levels of Cd, while the rest of the elements evidenced comparable levels with other localities. The higher levels of Cd in the tilapia can be explained by the bioavailability of this metal which may be associated with the mine tailing of the region; and the feeding habit of this fish, which is omnivorous benthic and also ingest detritus.

**Table 3:** Ranges and mean concentration (µg g^−1^ dw) of arsenic in tilapia worldwide

Species	As	Type of pollution	Region	References
*O. niloticus*		Agricultural and industrial	Manzala lake, Egypt	Sallam et al. (2019)
Muscle	3.45–3.87			
*O. niloticus*		Urban sewage and agriculture	Lake Phewam, Nepal	Rosseland et al. (2017)
Liver	1.0 (0.3–2.1)			
*O. niloticus*		Industrial	Koka lake, Ethiopia	Dsikowitzky et al. (2013)
Muscle	0.034–0.056			
Liver	0.077–0.568			
*O. niloticus*		Textile, ceramics, and municipal	Awasa lake, Ethiopia	Dsikowitzky et al. (2013)
Muscle	0.045–0.260			
Liver	0.267–0.437			
*O. niloticus*		Agriculture and wastewater	Tula river watershed, Mexico	Rubio-Franchini et al. (2016)
Muscle	0.018–0.089			
*S. melanotheron*		Agriculture and industrial	Awba dam, Nigeria	Adeogun et al. (2020)
Muscle	1.79			
*O. mossambicus*		As in groundwater	Farms SW coastal area Taiwan	Huang et al. (2003)
Muscle	0.858			
*O. mossambicus*		As in groundwater	Farms south of Taiwan	Lin et al. (2005)
Muscle	1.90 ± 1.31			
*O. mossambicus*		As in groundwater, industrial and agriculture	Farms west coast of Taiwan	Ling et al. (2013)
Muscle	8.57 ± 3.99			
*O. aureus*		Mine tailing spill (after 20 months)	El Comedero dam NW Mexico	This study
Muscle	0.09 ± 0.07			
Liver	1.55 ± 1.20			

– Not analyzed, moisture levels considered to change from wet weight to dry weight, muscle 83.2%, liver 80.5%, and guts 74.3% in viscera

**Table 4:** Ranges and mean concentration (µg g^−1^ dw) of cadmium, copper, and zinc in tilapia fish worldwide

Species	Cd	Cu	Zn	Type of pollution	Region	References
*O. niloticus*				Municipal	Yaounde lake, Cameroon	Léopold et al. (2015)
Muscle	0.11–0.23	0.59–4.11	15.4–47.2			
*O. niloticus*				Wastewater ponds	Wetland, East Calcutta, India	Chatterjee et al. (2016)
Liver	–	320	315			
*O. niloticus*				Mining towns	Kafue River, Zambia	Mbewe et al. (2016)
Muscle	0.30	2.8	*–*			
Liver	2.0	49.5	*–*			
*O. niloticus*				Sewage urban and agriculture	Lake Phewam, Nepal	Rosseland et al. (2017)
Liver	1.3 (0.4–1.8)	660 (120–988)	97 (61–132)			
*O. niloticus*				Agriculture, industrial, and urbanism	Mariut and Edku lakes, Egypt	Abdel-Moneim et al. (2016)
Liver	0.036–0.205	1.26–3.29	4.3–23.4			
*O. niloticus*				Industrial	Ologe lagoon, Owo and Etegbin River, Nigeria	Ndimele et al. (2017)
Muscle	*–*	34.7	10.5			
*O. niloticus*				Mining area	La Angostura dam, Sonora, Mexico	Martínez-Durazo et al. (2021)
Muscle	*–*	35.5 ± 10.0	18.7 ± 6.5			
Liver	*–*	649 ± 298	51.7 ± 10.1			
*O. niloticus*				Mining area	El Cajon de Onapa dam, Sonora, Mexico	Martínez-Durazo et al. (2021)
Muscle	*–*	18.5 ± 1.4	37.5 ± 7.3			
Liver	*–*	660 ± 393	46.5 ± 17.6			
*O. niloticus*				Mining area	El Oviachic dam, Sonora, Mexico	Martínez-Durazo et al. (2021)
Muscle	*–*	20.3 ± 3.0	17.1 ± 6.9			
Liver	*–*	521 ± 232	89.3 ± 28.9			
*Tilapia zillii*				Agriculture and industrial	Cross River, SE Nigeria	Okogwu et al. (2019)
Muscle	*–*	1.50	20.0			
*O. esculentus*				Agriculture and mining	Rukwa lake, Tanzania	Mapenzi et al. (2020)
Muscle	*–*	0.25–1.52	64.0–133.5			
*O. mossambicus*				Mining activities	Yonki dam, Papua New Guinea	Kapia et al. (2016)
Muscle	< 0.01	2.64	*–*			
*O. mossambicus*				Ponds influenced by domestic effluents	Malaysia	Yap et al. (2015)
Muscle	0.34–0.84	1.4–2.1	15.7–25.6			
Liver	1.28–3.05	8.9–269	53.5–101.9			
*O. mossambicus*				Lagoons influenced by agriculture and livestock	Valley Culiacan, NW Mexico	Izaguirre-Fierro et al. (1992)
Muscle	0.4–0.6	4.6–6.0	14–19			
*O. aureus*				Mining area	El Salto dam, NW Mexico	Frías-Espericueta et al. (2010)
Muscle	0.28 ± 0.03	0.98 ± 0.53	12.1 ± 2.6			
Liver	0.71 ± 0.31	147 ± 67	38.9 ± 12.1			
*O. aureus*				Mine tailing spill (after of 20 months)	El Comedero dam, NW Mexico	This study
Muscle	3.10 ± 0.94	1.48 ± 0.49	14.6 ± 4.1			
Liver	11.2 ± 8.5	871 ± 1261	89.6 ± 33.8			

– Not analyzed, moisture levels considered to change from wet weight to dry weight, muscle 83.2%, liver 80.5%, and guts 74.3% in viscera

Largemouth bass: The As concentrations in muscle (0.11 ± 0.23 µg g^−1^ dw) and liver (1.07 ± 0.48 µg g^−1^ dw) from EC dam were within the range reported in various regions of the world, where some type of pollution has been registered (Table 5). Similar to the tilapia and carp, the largemouth bass fish exhibited high Cd levels in muscle (1.70 ± 0.58 µg g^−1^ dw) and liver (3.90 ± 4.63 µg g^−1^ dw) compared to the compiled studies (Table 5), in which an oil spill, agriculture, and urbanism had been registered. Copper (1.00 ± 0.24 µg g^−1^ dw) and Zn (22.0 ± 4.8 µg g^−1^ dw) concentrations in the muscle of the largemouth bass samples of our study showed comparable or low levels to those registered in diverse studies worldwide (Table 5) where mining activity has been practiced. The liver of the largemouth bass from our study exhibited the highest level of Cu (390 ± 476 µg g^−1^ dw) and Zn (73.4 ± 10.1 µg g^−1^ dw) compared to those reported elsewhere (Table 5) associated with mining and agriculture activities.

**Table 5:** Ranges and mean concentration (µg g^−1^ dw) of arsenic, cadmium, copper, and zinc in the largemouth bass worldwide

Tissue	As	Cd	Cu	Zn	Type of pollution	Region	Reference
Muscle	0.1–0.3	0.1	0.6–1.0	11.2–21.1	Urban and oil spill	Reedy River watershe, South Carolina, USA	Otter et al. (2012)
Liver	0.1–0.3	0.3–0.7	6.5–48.6	47.1–87.3			
Muscle	0.78 ± 0.25	0.06 ± 0.03	1.08 ± 0.24	26.7 ± 3.90	Agriculture and industrial	Pearl River Delta, China	Leung et al. (2014)
Muscle	0.18 ± 0.00	0.06 ± 0.00	1.55 ± 0.18	–	Nuclear weapons production facility, USA	Savannah river, USA	Burger et al. (2002)
Muscle	0.041–1.181	–	–	–	Agricultural and industrial	Tablas de Daimile Nationa Park, Spain	Fernández-Trujillo et al. (2021)
Muscle	–	–	19.0–23.2	9.5–14.9	Mining area	La Angostura dam, Sonora, Mexico	Martínez-Durazo et al. (2021)
Liver	–	–	7.1–23.5	29.8–33.5			
Muscle	–	–	11.9–14.9	9.4–37.6	Mining area	El Cajon de Onapa, Sonora, Mexico	Martínez-Durazo et al. (2021)
Liver	–	–	7.1–11.9	11.9–32.7			
Muscle	–	–	13.0–15.5	11.9–15.3	Mining area	El Oviachic dam, Sonora, Mexico	Martínez-Durazo et al. (2021)
Liver	–	–	7.5–12.2	48.2–58.5			
Muscle	0.11 ± 0.23	1.70 ± 0.58	1.00 ± 0.24	22.0 ± 4.8	Mine tailing spill (after of 20 months)	El Comedero dam, NW Mexico	This study
Liver	1.07 ± 0.48	3.90 ± 4.63	390 ± 476	73.4 ± 10.1			

– Not analyzed, moisture levels considered to change from wet weight to dry weight, muscle 83.2%, liver 80.5%, and guts 74.3% in viscera

Although regional comparisons constitute a robust approach to examining concentration levels in organisms from different regions due to exclusion of various variables involved (size, age, sex, etc.), this exercise allows us to generalize that the three fish species are exposed to high levels of bioavailable Cd in EC dam. In the particular case of the largemouth bass, which is predominantly piscivore, it is evident that Cu and Zn are highly accumulated in the liver, also revealing an elevated bioavailable fraction for these two metals in EC dam.

### Metal(loid)s in muscle, food safety guidelines, and risk assessment

In the context of human health by the consumption of the edible fraction of fish, the muscle is frequently the focus since it is the main support of the human diet (Bergés-Tiznado et al., 2015). The local human population consumes the three fish species, but mainly tilapia fillet, with an average consumption per capita in Mexico of 3.08 kg (FAO, 2021). Regarding As, mean concentrations in the muscle of the three fish species were below (< 0.03 µg g^−1^, wet weight) the maximum permissible limit (MPL) considered in the Mexican legislation (80 µg g^−1^ ww, DOF, 2011) for fish and seafood. The legal Cd MPL in Mexico (DOF, 2011) and internationally (FAO, 1983) is 0.5 µg g^−1^ (ww) in muscle and no individual of the common carp and largemouth bass had concentrations above this value (Fig. 2; Fig. 2SM); in contrast, the blue tilapia exhibited an average (0.62 ± 0.21 µg g^−1^ ww) exceeding the MPL (64% of individuals). Cu and Zn are not considered in the Mexican guidelines. Nonetheless, countries such as Australia and India established a MPL of 10 µg g^−1^ ww for Cu, and 40 µg g^−1^ ww for Zn in New Zealand (FAO, 1983); the FAO/WHO (1989), and the European Union (MAFF, 2000) use a MPL of 30 and 20 µg g^−1^ ww for Cu, and 40 and 50 µg g^−1^ ww for Zn, respectively. However, no specimen exhibited levels in the muscle above such limits (Fig. 2; Fig. 2SM). Evidently, fishes collected after a mine-tailing spill should be considered with extreme caution when taken for human consumption, and other elements potentially associated with mine-tailing should be examined as well. This study shows that the element concentrations quantified in the edible portion, with the partial exception of Cd, are low and below the MPL (Fig. 2; Fig. 2SM).

The calculated risk assessments from the first scenario into a specific ration of consumption according to each fish species were all THQ’s values < 1 and the sums of the individual hazard quotients as HI were also lower than 0.1 in all the population considered (Table 6). These results indicate the consumption of a meal of 15 g of tilapia, 3 g of common carp, and 6.3 g of largemouth bass in a week is harmless and there will be no risk of adverse health effect. If an intake of 200 g week^−1^ of each one of the three species is consumed (Table 6), there might be risk from Cd in children feeding on muscle of blue tilapia due its HI is nearly 1, so attention must be paid in this population strata. All the others THQ’s and HI’s were < 1, meaning there will be no risk of consuming common carp nor largemouth bass.

**Table 6:** Non-cancer risk assessment by population group from specific and total fish per capita rations for blue tilapia, common carp and largemouth bass; children BW = 20 kg, women BW = 65 kg, and men BW = 75 kg

Element	THQ blue tilapia (15 g week^−1^)			THQ common carp (3 g week^−1^)			THQ largemouth bass (6.3 g week^−1^)		
	Children	Women	Men	Children	Women	Men	Children	Women	Men
As	0.007	0.002	0.002	0.001	< 0.001	< 0.001	0.005	0.001	0.001
Cd	0.066	0.020	0.018	0.004	0.001	0.001	0.019	0.006	0.005
Zn	0.001	< 0.001	< 0.001	< 0.001	< 0.001	< 0.001	0.001	< 0.001	< 0.001
HI	0.075	0.023	0.020	0.006	0.002	0.002	0.024	0.007	0.006

Element	THQ blue tilapia (200 g week^−1^)			THQ common carp (200 g week^−1^)			THQ largemouth bass (200 g week^−1^)		
As	0.095	0.029	0.025	0.095	0.029	0.025	0.143	0.044	0.038
Cd	0.888	0.273	0.237	0.329	0.101	0.088	0.601	0.185	0.160
Zn	0.014	0.004	0.004	0.020	0.006	0.005	0.025	0.008	0.007
HI	0.997	0.307	0.266	0.444	0.137	0.118	0.770	0.237	0.205

By using PTI’s data to estimate a safe intake of the three species according to every measured element, it could be noted that estimates for essential elements like Cu and Zn presented extremely high consumption rates to be harmful to human health. For example, children must consume 2.1 and 37.1 kg day^−1^ of blue tilapia to be at risk from Zn and Cu, respectively. For a 70 kg BW person the intake must be of 7.2 and 129.6 kg day^−1^, respectively. These intake rates are quite unreal to be consumed and were similar to the data from common carp and largemouth bass, even higher in some cases. But for the non-essential elements the intake rations decreased considerably compared to Cu and Zn. The levels of As in the three species were very low (< 0.03 µg g^−1^) but considered in its inorganic form, the consumption weekly rates for blue tilapia and common carp were 700 g for children and higher for woman and men (2.3 and 2.6 kg). The intake recommended for not to be in risk from Asi exposure if largemouth bass filet is consumed would be 500 g for children and, also higher for woman and men (1.5 and 1.8 kg). The intake rates decreased when Cd was involved, especially in the blue tilapia muscle; children must consume a maximum of 200 g week^−1^ to avoid adverse health effects by Cd, instead men and women could consume up to 700 and 800 g week^−1^, respectively, for not be at risk. Finally, the proposed weekly safe intake for children feeding on common carp is to consume no more than 500 g, and for men and woman up to 1.8 kg and when the meal is muscle of largemouth it is recommended a ration under 300 g for children and 1 kg for men and women.

## Conclusions

The accumulation patterns in the tissues were different in the fish species and metal(loid)s, which confirm the first hypothesis, in which was proposed that the three fish species with different feeding habit exhibit variable metal(loid)s accumulation. In the three fish species, Cd and Cu concentrations were consistently higher in the liver than in other tissues, while Zn concentration showed a different pattern; in the tilapia, the liver had the highest level, and in the carp and largemouth bass, the gills exhibited higher concentrations. Arsenic showed a different pattern that was consistent in the three fish species, with higher levels in the guts. Such variability can be caused mainly by the distinct feeding habit of each fish species. Tilapias consume a heterogeneous diet (benthic organisms, plankton, and detritus); therefore, high element concentrations found in its tissues and their bioconcentration could be associated with the ingestion of detritus, including residues of the mine-tailing spill.

Compared to a study carried out 90 days after the mine spill during a massive mortality of tilapia in EC dam (Pàez-Osuna et al., 2022), metal(loid)s levels decreased 129, 5, 10, and 1.7 times for As, Cd, Cu, and Zn, respectively, 20 months after the spill (present study), which confirms the second hypothesis stated in the present study, in which a reduction of the metal(loid) concentrations in fish should be reached after the mine spill. This robust comparison reveals that cleaning operations were more efficient for As and less for Cu, Cd and Zn. Furthermore, concentrations in this study were ~ 16, ~ 6, and ~ 2 times higher for Cd, Cu, and Zn, respectively, compared to those registered in the liver of tilapia studied in a dam (~ 30 km from EC dam; 2004–2005) affected by agriculture. These results indicate that tilapia from this study accumulated higher levels than those observed previously in the region.

### Supplementary Information

Below is the link to the electronic supplementary material.

Supplementary file1 (DOC 118 KB)

## Data Availability

The datasets used and/or analyzed during the current study are available from the corresponding author on reasonable request.
