# Supplementary material for: Metal(loid)s (As, Cd, Cu, and Zn) in three fish species from a dam after a mine-tailing spill: differential bioaccumulation and potential health risk
Source: Environ Geochem Health. 2023 Feb 28;45(7):4533–48. doi: 10.1007/s10653-023-01509-8 (PMC10310605; doi:10.1007/s10653-023-01509-8)
Supplement: Supplementary file 1 — Supplementary file1 (DOC 118 KB) [file 10653_2023_1509_MOESM1_ESM.doc]

**Supplementary material of the manuscript**

Article title:

Metal(loid)s (As, Cd, Cu, Zn) in three fish species from a dam after a mine-tailing spill: differential bioaccumulation and potential health risk

Journal name: Environmental Geochemistry and Health

Author names: F. Páez-Osuna *, M.E. Bergés-Tiznado, G. Valencia-Castañeda, M.G. Fregoso-López, J.A. León-Cañedo, J.F. Fierro-Sañudo, J. Ramírez-Rochín

Affiliation and e-mail address of the corresponding author:

Universidad Nacional Autónoma de México, Instituto de Ciencias del Mar y

Limnología, Unidad Académica Mazatlán. P.O. Box 811, Mazatlán 82000, Sinaloa,

Mexico. ORCID: 0000-0002-1579-817X

Miembro de El Colegio de Sinaloa, Antonio Rosales 435 Poniente, Culiacán, Sinaloa,

Mexico

paezos@ola.icmyl.unam.mx

**Table 1SM.** Measured and certified values and recoveries (±SD) of Standard Reference Material DORM-4 (n=9), including the detection limit (DL)

| Element | Certified  (mg kg^-1^) | Measured  (mg kg^-1^) | Recovery  (%) | DL  (µg L^-1^) | CV  (%) |
| --- | --- | --- | --- | --- | --- |
| Cu | 15.9 ± 0.9 | 14.7 ± 1.2 | 92.2 ± 7.6 | 0.015 | 0.7 |
| Zn | 52.2 ± 3.2 | 55.3 ± 4.5 | 105.9 ± 8.6 | 0.013 | 1.3 |
| Cd | 0.306 ± 0.015 | 0.29 ± 0.03 | 93.5 ± 11.2 | 0.054 | 5.2 |
| As | 6.80 ± 0.64 | 6.50 ± 0.20 | 95.0 ± 3.5 | 0.350 | 1.8 |

SD= standard deviation, n= sample number, CV= coefficient of variation

**Table 2SM.** Correlations among morphometric variables and levels of As, Cd, Cu, and Zn in the studied tissues of the largemouth bass (p<0.05)

| Element/tissue |  | Total length (cm) |  | Weight (g) |
| --- | --- | --- | --- | --- |
| As_muscle_ |  | -0.69 (p= 0.003) |  | -0.66 (p= 0.006) |
| As_liver_ |  | NS |  | NS |
| As_gills_ |  | 0.54 (p= 0.030) |  | 0.55 (p= 0.028) |
| As_guts_ |  | NS |  | NS |
| Cd_muscle_ |  | 0.78 (p< 0.001) |  | 0.81 (p< 0.001) |
| Cd_liver_ |  | NS |  | NS |
| Cd_gills_ |  | 0.69 (p= 0.003) |  | 0.71 (p= 0.002) |
| Cd_guts_ |  | 0.53 (p= 0.034) |  | 0.60 (p= 0.015) |
| Cu_muscle_ |  | NS |  | NS |
| Cu_liver_ |  | NS |  | NS |
| Cu_gills_ |  | NS |  | NS |
| Cu_guts_ |  | NS |  | NS |
| Zn_muscle_ |  | NS |  | NS |
| Zn_liver_ |  | NS |  | NS |
| Zn_gills_ |  | NS |  | -0.50 (p= 0.049) |
| Zn_guts_ |  | -0.75 (p= 0.001) |  | -0.76 (p= 0.001) |

NS= not significant (p>0.05)

Figure 1SM. Variation of the concentration of Cu in liver of the common carp versus the total length (TL) and the weight.


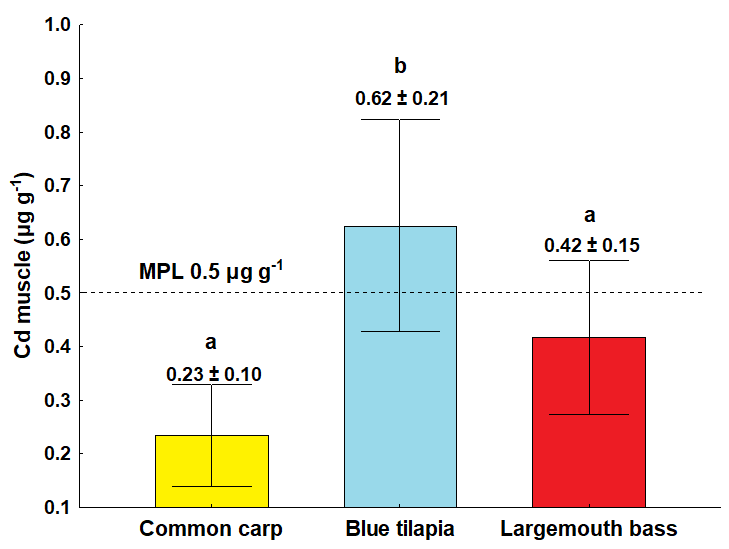

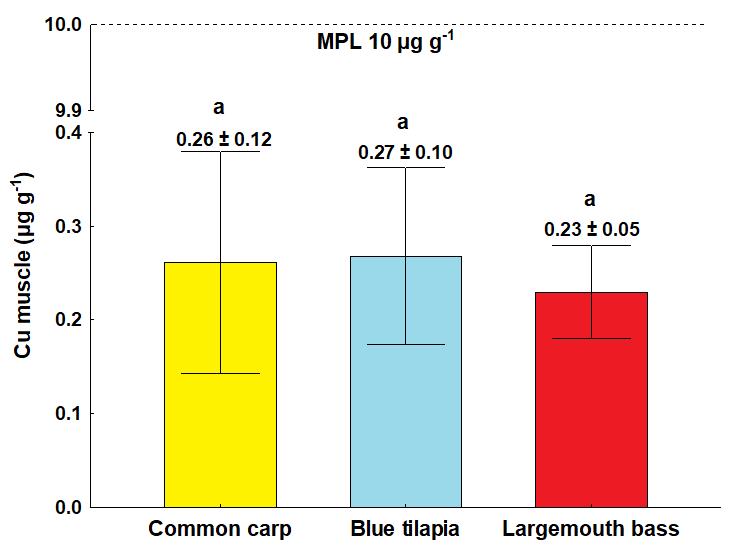


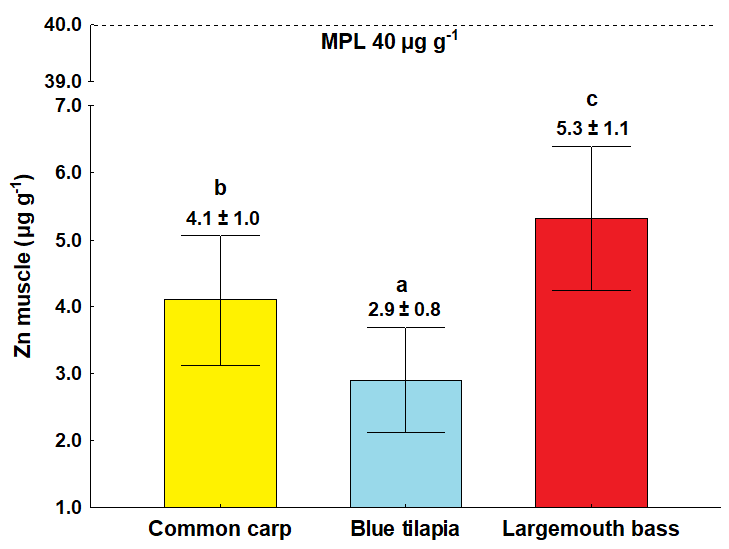


Figure 2SM. Metal mean (μg g^-1^±SD; wet weight) concentrations in the muscle of the common carp, blue tilapia, and largemouth bass; maximum permissible limit (MPL; dotted lines); different letters above bars indicate significant differences (p<0.05) between means of the fish species.
